# Treatment of hepatitis C genotype 1 patients with severe fibrosis or compensated cirrhosis: the telaprevir early access program in patients from Romania

**DOI:** 10.1186/1471-2334-13-S1-P58

**Published:** 2013-12-16

**Authors:** Adrian Streinu-Cercel, Anca Victorița Trifan, Florin Alexandru Căruntu, Ioan Sporea, Liliana Simona Gheorghe, Manuela Curescu, Mihai Mircea Diculescu, Mihai Voiculescu, Oliviu Pascu, Isabelle Lonjon-Domanec, Andrew Martin Hill, Sorin Rugină

**Affiliations:** 1 Carol Davila University of Medicine and Pharmacy, Bucharest, Romania; 2 National Institute for Infectious Diseases “Prof.Dr. Matei Balş”, Bucharest, Romania; 3 “Gr.T.Popa” University of Medicine and Pharmacy, Iaşi, Romania; 4 Institute of Gastroenterology and Hepatology, “St Spiridon” Emergency Hospital, Iaşi, Romania; 5 Victor Babeş University of Medicine and Pharmacy, Timişoara, Romania; 6 Center for Digestive Diseases and Liver Transplantation, Fundeni Clinical Institute, Bucharest, Romania; 7 Regional Institute of Gastroenterology and Hepatology, Cluj-Napoca, Romania; 8 Janssen Pharmaceuticals, Paris, France; 9 MetaVirology Ltd, London, UK; 10 Ovidius University, Constanța, Romania; 11 Clinical Hospital of Infectious Diseases, Constanța, Romania

## Background

HEP3002 is an ongoing, open-label, early access program of telaprevir in 16 countries, for patients with genotype 1 hepatitis C with severe fibrosis or compensated cirrhosis. This interim analysis is of 16 week data from the 209 patients from Romania.

## Methods

Patients were treated with telaprevir in combination with peginterferon alfa and ribavirin (PR) for 12 weeks, followed by PR for 12 or 36 weeks. Severe fibrosis/cirrhosis was defined by liver biopsy (Metavir F3-4 or Ishak 3-6) or non-invasive tests. Platelet count >90,000/cmm was required at entry. HCV RNA was evaluated at baseline and Weeks 4 and 12 of treatment. Virological response was defined as serum HCV RNA not detected, for the Intent to Treat (ITT) population.

## Results

Mean age was 52 years; 47% were male and 100% Caucasian, 59% had HCV RNA levels ≥800,000 IU/mL, 58%/42% had severe fibrosis/cirrhosis, 2% had genotype 1a, 14% were treatment naïve, 75% prior relapsers, 3% prior partial responders, 7% prior null responders and 1% had prior viral breakthrough. HCV RNA responses (percent undetectable) at weeks 4 and 12 (ITT analysis) are shown in Table 1.

**Table 1:** 

Percent HCV RNA not detected	Week 4 (RVR)	Week 4+12 (eRVR)	Week 12
Treatment-naïve (n=30)	73%	70%	93%
Prior relapser (n=156)	85%	81%	94%
Prior partial responder (n=6)	100%	83%	83%
Prior null responder (n=15)	80%	73%	80%
Overall^a^ (n=209)	83%	79%	92%

^a^includes 2 patients with prior virological break-through, not in four categories above

Nine patients (4%) discontinued TVR due to adverse events, including six (3%) for rash and one (<1%) for anaemia. The rate of serious adverse events was 9% and no patients died during the study.

## Conclusion

In this telaprevir early access program for hard-to-cure patients with severe fibrosis or compensated cirrhosis, early on-treatment virological responses are encouraging. Rates of discontinuation of telaprevir for adverse events were similar to Phase 3 trials.

